# Elastic net-based prediction of IFN-β treatment response of patients with multiple sclerosis using time series microarray gene expression profiles

**DOI:** 10.1038/s41598-018-38441-2

**Published:** 2019-02-12

**Authors:** Arika Fukushima, Masahiro Sugimoto, Satoru Hiwa, Tomoyuki Hiroyasu

**Affiliations:** 1 Doshisha University, Graduate School of Life and Medical Sciences, Kyoto, Japan; 2 Research and Development Center for Minimally Invasive Therapies Health Promotion and Preemptive Medicine, Tokyo Medical University, Shinjuku, Tokyo 160-8402 Japan; 3 Institute for Advanced Biosciences, Keio University, Tsuruoka, Yamagata 997-0052 Japan; 4 University of Tsukuba, Research and Development Center for Precision Medicine, Tukuba, Ibaraki 305-8550 Japan

## Abstract

INF-β has been widely used to treat patients with multiple sclerosis (MS) in relapse. Accurate prediction of treatment response is important for effective personalization of treatment. Microarray data have been frequently used to discover new genes and to predict treatment responses. However, conventional analytical methods suffer from three difficulties: high-dimensionality of datasets; high degree of multi-collinearity; and achieving gene identification in time-course data. The use of Elastic net, a sparse modelling method, would decrease the first two issues; however, Elastic net is currently unable to solve these three issues simultaneously. Here, we improved Elastic net to accommodate time-course data analyses. Numerical experiments were conducted using two time-course microarray datasets derived from peripheral blood mononuclear cells collected from patients with MS. The proposed methods successfully identified genes showing a high predictive ability for INF-β treatment response. Bootstrap sampling resulted in an 81% and 78% accuracy for each dataset, which was significantly higher than the 71% and 73% accuracy obtained using conventional methods. Our methods selected genes showing consistent differentiation throughout all time-courses. These genes are expected to provide new predictive biomarkers that can influence INF-β treatment for MS patients.

## Introduction

Multiple sclerosis (MS) is one of the most common neurological disabilities of the central nervous system^1^. The highest incidences of MS have been reported in North America and Europe (100/100,000), and the lowest occur in East Asia and sub-Saharan Africa (2/100,000)^2^. This disease is the second most common neurological disability in young adulthood^3^. Approximately 80–90% of MS patients initially suffer from relapsing-remitting MS (RRMS) where MS repeatedly occurs with a variety of symptoms, including the stages of neurological disability (relapse) and recovery (remission)^1^. The disease gradually shifts to secondary progressive MS (SPMS) which is associated with frequent relapses. Therefore, a systematic treatment strategy to prevent and/or delay relapse is important for the improvement of the quality of life (QOL) of MS patients.

Interferon-β (INF-β) has been commonly used to prevent relapse of MS^4,5^ however, INF-β treatment has two issues. First, the treatment only works for a limited number of patients, where approximately half of the patients relapse within 2 years despite treatment^6,7^. Second, this treatment can cause side effects, such as spasticity and dermal reaction^5^. Thus, effective surveillance and appropriate intervention over a long period of time post-treatment is required. Although the pathogenesis of MS has yet to be fully elucidated, various genetic factors involved in this disease have been reported^8^. Gene expression data have been intensively analyzed to predict INF-β treatment responses^4,5,8–12^. Hundreds of genes, such as *Caspase2*, *Caspase*1*0*, and *FLIP*, showed promise in predicting treatment response^5,11^; however, these genes were identified by conventional statistical methods which showed low prediction accuracies in some cases^11,12^. The *MxA* and *ISG* genes were reported to be predictive for IFN-β treatment response^8–10^. The expression patterns of these genes, however, were not consistently differentiated throughout all of the time-courses. Given this, any predictions would only be accurate immediately after the observation of the gene expression levels, while the accuracy of prediction would be low for subsequent responses^9^. Therefore, the identification of genes showing highly accurate prediction abilities throughout all time-courses is needed.

**Figure 1:**
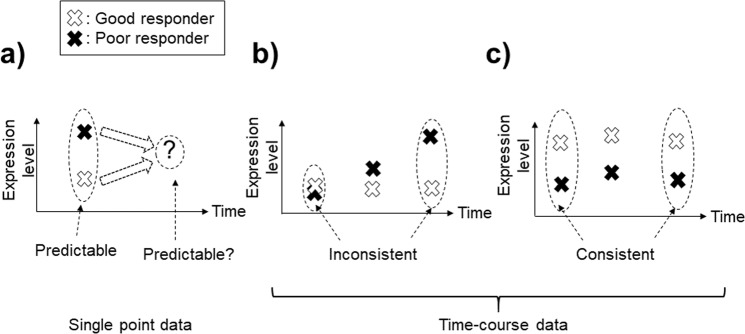
The concepts of prediction using gene expression data. (**a**) Genes identified by single time-point data. (**b**) Genes showing inconsistent differentiation between the current and the future time-points. (**c**) Genes showing consistent differentiation throughout the data across multiple time-points.

Generally, data analyses to identify biomarkers are categorized into single time-point and time-course-based approaches^13,14^. Prediction using only the currently observable data to predict an outcome of treatment is the most useful but most challenging approach for optimizing patient treatment. Single time-point-based analyses^13^ (Fig. 1a) are challenging because the gene expression levels observed during the progression of MS are dynamic^14–16^. Prediction using time-course data consisting of multiple time-points would result in more accurate predictions^15,17–22^ by eliminating the selection of genes showing inconsistent differentiation throughout the observation period (Fig. 1b,c). In particular, the identification of genes showing highly accurate prediction abilities throughout all time-courses is important for highly accurate prediction (Fig. 1c; this scenario is termed the “static-longitudinal scenario”)^22^. Microarray data analyses present several difficulties, including the problem of high-dimensionality (a higher number of genes compared to sample size) and the high degree of multi-collinearity. Elastic net, a type of sparse modelling method, has been commonly utilized to identify differentiated genes to address these issues^15,22–25^. To our knowledge, however, the identification of genes showing highly accurate prediction abilities throughout all time-courses for MS patients by sparse modelling has not been reported.

The purpose of this study was to identify new genes showing highly accurate prediction abilities throughout all time-courses for MS patients. Therefore, sparse modelling methods were modified, and two microarray time-course datasets collected from patients with MS were used for predictions of INF-β treatment responses by our proposed method.

## Methods

Elastic net^25^, a sparse modelling method, was modified to analyse time-course data. Our method was designed to find genes showing consistent differentiation between the two given groups throughout multiple time-points. Here, we addressed the following problems:

1. High dimensionality. Microarray data includes a larger number of genes compared to a small sample size.
2. Multi-collinearity. Microarray data includes many genes showing highly positive correlations. The use of these genes for a prediction model would deteriorate generalization ability^26^.
3. Time-courses. Genes showing consistent differentiation throughout multiple time-points should be identified.

Elastic net was designed to analyse single time-point data to identify differentiated genes by preventing multi-collinearity^25^. We modified this method for the time-course data analyses.

### Elastic net and stability selection

Sparse modelling is one of a variety of selection methods suitable for high dimensional data analyses^25,27^. Among the different sparse modelling methods, Least Absolute Shrinkage and Selection Operator (LASSO)^28^ have been commonly used in various studies^15,23,24^. LASSO, however, is limited in that it selects only one variable from two variables showing a high correlation (multicollinearity), and the other variables are not selected despite being differentiated^25^. The Ridge regression model is a method capable of solving this problem^29^. This method can construct models from two variables showing a multi-collinearity; however, this method does not select genes. Elastic net is another sparse modelling method able to reduce those two limitations. Elastic net is comprised of LASSO and Ridge^26^, which selects variable sets, and this method selects all variables, even those showing high multi-collinearities^26,30,31^. Here, we employed Elastic net rather than LASSO to select gene candidates showing predictive abilities for subsequent analyses.

The proposed method used a logistic regression (eq. 1) to predict the INF-β treatment response based on differentiated genes^15,30,32^.

1 $${\rm{\Pr }}\,({\rm{y}}=1|{{\boldsymbol{X}}}_{{\boldsymbol{t}}})=\frac{1}{1+{e}^{-({{\boldsymbol{X}}}_{{\boldsymbol{t}}}{\boldsymbol{\beta }})}}$$

where, **y** = {*y*_1_, *y*_2_, …, *y*_*n*_; *y*_*i*_ ∈ {0, 1}}: *y*_*i*_ denotes the response variable that included good responders (labelled as 1) or poor responders (labelled as 0) to INF-β treatment, respectively. *n* denotes the sample size of MS patients. ${{\boldsymbol{X}}}_{{\boldsymbol{t}}}=\{{{\bf{x}}}_{{\boldsymbol{t}},1},{{\bf{x}}}_{{\boldsymbol{t}},2},\ldots ,{{\bf{x}}}_{{\boldsymbol{t}},{\boldsymbol{p}}};\,{{\bf{x}}}_{{\boldsymbol{t}},{\boldsymbol{j}}}^{\top }=\{{x}_{t,j}^{(1)},{x}_{t,j}^{(2)},\ldots ,{x}_{t,j}^{(n)};\,j=1,\ldots ,p\}\}$: ***X***_***t***_ denotes the explanatory variables of gene expression levels at time-point *t*, and *p* denotes the number of genes. ***β*** = {*β*_1_, *β*_2_, …, *β*_*p*_}: ***β*** denotes the regression coefficients.

The regression coefficient ***β*** in eq. 1 indicates the degree of association between the response to INF-β treatment and each gene. Therefore, a gene with a high absolute value of a regression coefficient was selected as a gene bearing the predictive ability of the treatment response. Regression coefficients, however, were difficult to calculate by Ordinary Least Squares (OLS), a general method for calculation of the regression coefficients, due to the high dimensionality of the microarray data (the number of genes *p* ≫ the sample size *n*). Therefore, a small number of differentiated genes should be selected prior to the use of OLS. Sparse modelling assumes that only several regression coefficients are needed for the prediction model and that the others are not needed. This assumption means that the regression coefficient values of several genes which were needed for the prediction model were non-zero while the other values were zero. Specifically, genes exhibiting non-zero regression coefficients were selected as genes able to predict responses to INF-β treatment. With the use of Elastic net, regression coefficients were calculated by adding a penalty term to a least-square loss function (eq. 2).

2 $$\mathop{{\rm{argmin}}}\limits_{{\boldsymbol{\beta }}}\,{\rm{J}}({\boldsymbol{y}},{{\boldsymbol{X}}}_{{\boldsymbol{t}}})+\lambda \,\sum _{j=1}^{p}\,{w}_{j}[(1-\alpha )\frac{1}{2}{\beta }_{j}^{2}+\alpha |{\beta }_{j}|]$$

where, J(***y***, ***X***_***t***_) denotes loss of function of OLS, and λ denotes the hyper-parameter for the penalty term of Elastic net. The penalty term was given after the second term of the equation. Hyper-parameters were generally set by analysts. *α* (0 ≤ α ≤ 1) denotes the hyper-parameter that indicated the degree between the Ridge $(\frac{1}{2}{\beta }_{j}^{2})$ and LASSO ($|{\beta }_{j}|$) terms. ***w*** = {*w*_*t*,1_, *w*_*t*,2_, …, *w*_*t*,*p*_} (${\boldsymbol{w}}\in {{\mathbb{R}}}_{ > 0}$): ***w*** denote the weights of Elastic net as the selection bias of each gene at a given time-point *t*. Thus, genes showing a larger or a lower weight were selected at a lower or a higher probability, respectively.

Cross validation is commonly used for optimizing the λ value in eq. 2. Inconsistent genes, however, are generally selected depending on the λ value. To prevent this problem, Stability Selection (SS)^33^ was used. SS selects for genes according to the following procedures:

1. A subset of samples was obtained from the gene expression data by random sampling.
2. An arbitrary λ value was provided to Elastic net to select genes using the data of (1).
3. (1–2) were repeated with multiple subsets.
4. The frequency of selection using an arbitrary λ value was calculated for multiple subsets.
5. (1–3) were repeated using multiple λ values.
6. For each gene, the maximum of the probability calculated in (4) among multiple λ values was regarded as the selection probability of the gene.
7. Genes showing a selection probability above the threshold θ_*ss*_ were selected.

### Proposed method: marker identification using time-course data

**Figure 2:**
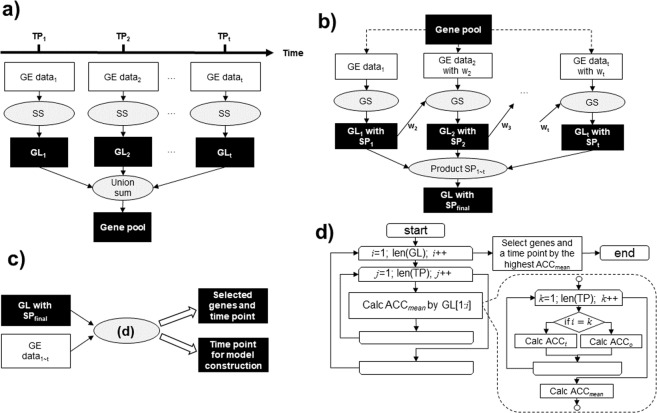
The concept of the proposed method. (**a**) Creating the gene pool by SS using gene expression data at each time-point. (**b**) Gene selection (GS) using candidate genes and calculating selected probability (SP). GS used Elastic net with SS assigning weights (w_1~t_) to gene expression data at each time-point. (**c**) Identifying the genes from gene lists (GLs) with SP. (**d**) The flow of the third step in the proposed method. In this step, prediction models were evaluated by using LOO (time-point for model construction = time-point for prediction) and utilizing different data (time-point for model construction ≠ time-point for prediction).

The proposed method consisted of the following three procedures (Fig. 2):

1. Screening of gene candidates (Fig. 2a). Due to the difficulties associated with high dimensional problems, Elastic net along with SS was used for the screening of gene candidates, known as the gene pool, from the data at each time-point. Only genes selected at least one time by Elastic net using SS were selected in the gene pool and the rest were eliminated.
2. Ranking of genes showing consistent differentiation throughout multiple time-points (Fig. 2b). Modified Elastic net was used to select genes showing consistent differentiation throughout multiple time-points from the gene pool. Initially, Elastic net incorporating SS selected predictive genes from the gene pool at the first time-point. Then, at the next time-point *t*, Elastic net (eq. 2) using SS was conducted with a higher selection bias to select genes which were selected at the previous time-point *t* − 1. Therefore, Elastic net sets the weights of genes selected at *t* − 1 to values smaller than genes not selected (eq. 3). This procedure was repeatedly performed at subsequent time-points. Consequently, genes showing consistent differentiation throughout multiple time-points were identified using the following:
  3 $${w}_{t,j}=\{\begin{array}{lll}1,\,{\rm{if}}\,{g}_{j} & \in  & G{L}_{t-1}\\ {\rm{\gamma }},\,{\rm{if}}\,{g}_{j} & \notin  & G{L}_{t-1}\end{array}$$
  where *w*_*t*,*j*_ denotes the weight of the *j*^*th*^ gene in Elastic net at *t* in eq. 2, ${\rm{\gamma }}({\rm{\gamma }}\in {{\mathbb{R}}}_{ > 0};\,{\rm{\gamma }} > 1)$ denotes the selection bias; *g*_*j*_ denotes the *j*^*th*^ gene, and *GL*_*t*−1_ denotes a gene list at *t* − 1. The gene list was constructed using selected genes at *t* − *1*.
  Finally, the product ***SP***_***final***_ was calculated by selection probability at each time-point for each gene, and the genes were ranked in descending order according to selection probabilities. The product ***SP***_***final***_ denoted the probabilities based on the frequency of selection of each gene throughout all time-points (eq. 4). The product ***SP***_***final***_ was ranked in descending order. According to this ranking, the gene list for the prediction model was created for use in the third step of the model using the following:
  4 $$\begin{array}{rcl}{\boldsymbol{S}}{{\boldsymbol{P}}}_{{\boldsymbol{final}}} & = & \{S{P}_{1},\,S{P}_{2},\ldots ,\,S{P}_{p}\,\}\\ S{P}_{j} & = & \,\prod _{t=1}^{T}\,S{P}_{t,j}\end{array}$$
  where *SP*_*t*,*j*_ denotes selection probability of the *j*^*th*^ gene by stability selection at *t*.
3. Construction of a prediction model using the ranked genes (Fig. 2c). Genes for the prediction model were identified based on the gene list ranked in the second step. The time-point for data to be used for constructing the prediction model was also selected simultaneously. Here, prediction models of treatment response were constructed using combinations of various groups of genes and time-points of gene expression data. To identify the genes and select a time-point of data for the prediction model, these models were evaluated (Fig. 2d). An evaluation value was calculated by the prediction model that was constructed by one group of genes using time-point data. The genes in the group with the best evaluation value were identified as the genes showing consistent differentiation. These time-point data were selected for the prediction model. This group of genes was created by individually adding genes from the gene list generated in the second step in descending order. The prediction models of all gene groups were constructed and evaluated at each time-point of the time-course data.

In this step, prediction accuracy, a ratio describing the prediction accuracy of model data against data not used for constructing the model, was used as an evaluation value. A prediction model was constructed from a group of genes using data at a given time-point, and the prediction accuracy was calculated. Prediction accuracies were calculated at each time-point for model construction, as shown in the following two cases.

#### Case 1: Time-point for model construction = time-point for prediction

A constructed model was used for the prediction of data at identical time-points. Leave-one-out (LOO) was used to evaluate the prediction accuracy (ACC_*o*_). In LOO, one sample of data was used as test data, and other data were used for model construction. LOO was repeated until all the samples became test data.

#### Case 2: Time-point for model construction ≠ time-point for prediction

A constructed model was used for the prediction of the data at a time-point not used for model construction. The prediction accuracies (ACC_*T*_) were calculated using the data at time-points for prediction.

The mean of prediction accuracies (ACC_*mean*_) was calculated for each group of genes and for each time-point of model construction in eq. 5.

5 $${{\rm{ACC}}}_{mean}=\frac{1}{T}(\sum _{d\,\in \,{\boldsymbol{D}}}\,AC{C}_{T}^{(d)}+AC{C}_{o})$$

where ACC_*mean*_ denotes the mean of the prediction accuracy for the prediction model constructed by a group of genes and a time-point of data. $T$ denotes the length of all time-points. $AC{C}_{T}^{(d)}$ denotes the prediction accuracy using data at *d*. ***D*** = {*t*_1_, *t*_*t*_, …, *t*_*T*_} denotes the time-points of ACC_*T*_. *t* was not included for the time-point used for model construction. ACC_*o*_ denotes the prediction accuracy using data at the time-point used for model construction.

The genes in the group with the best ACC_*mean*_ were identified as the selected genes. This model was constructed using the data from a given time-point. This time-point was selected as the time-point for model construction.

### Numerical experiments

The prediction accuracies of the developed models were evaluated for the prediction of INF-β treatment responses. The prediction accuracies of the proposed and conventional methods were compared.

#### Material and pre-processing

The evaluated data consisted of the time-course gene expression data from two MS patients who underwent INF-β treatment. The two datasets of GSE24427 (Dataset A)^34^ and GSE19285 (Dataset B)^35^ were used. These datasets included time from start of therapy to the first relapse; however, the definition of response is different for the two datasets^34,35^. Table 1 shows the number of time-course points in each data platform, and the method of normalization. Log_2_-fold change and quantile normalization were performed for pre-processing of gene expression data. Subsequently, the expression levels of each gene were converted to *Z*-scores.

**Table 1:** Summary of gene expression datasets of INF-β treatments for MS patients.

Name of dataset	Dataset A	Dataset B
GEO ID	GSE19285	GSE24427
Type of INF-β	Intramuscular Interferon beta 1a	Subcutaneous Interferon beta 1a
Time-points	first (t1), Second (t2), fifth (t3)	first (t1), Second (t2), 1 month (t3) 12 month (t4), 24 month (t5)
Number of good responders	15	16
Number of poor responders	9	9
Number of genes	11220	13513
Gene expression	Peripheral blood mononuclear cells	Peripheral blood mononuclear cells
Platform	Affymetrix Human Genome U133A Array	Affymetrix Human Genome U133A Array
Preprocessing for microarray	MAS5.0	MAS5.0

In this paper, symbols for time-points were presented as “t1”, “t2”, “t3”, etc.

#### Conventional method

The conventional method used only for the gene expression data at a single time-point. Elastic net with SS using data at a single time-point was used as the conventional method. Genes were ranked according to the selection probabilities by SS. Finally, using the procedures of the proposed method (Fig. 2d), ACC_*mean*_ was calculated using these selection probabilities. Thereafter, the genes in the group with the best ACC_*mean*_ were regarded as identified genes. These genes were regarded as genes with the best performance throughout multiple time-points in the conventional method using data from a single time-point.

### Evaluation method

The prediction accuracies were calculated by eq. 6. These were calculated using only test data which were not used for model construction. To evaluate the prediction accuracies using the data at the time-point used for model construction, LOO was conducted. To evaluate the prediction accuracies using the data at the other time-points, all available data were used.

6 $${\rm{ACC}}\,[ \% ]=\frac{TP+TN}{TP+FP+FN+TN}\,\ast \,100$$

where *TP* denotes the number of true positives, *FP* denotes the number of false positives, *FN* denotes the number of false negatives, and *TN* denotes the number of true negatives in the test data.

First, the prediction accuracies of the construction models were evaluated. In order to compare the prediction model of the proposed and the conventional method, the mean prediction accuracy (ACC_*mean*_) throughout all time-points was calculated using ACC_*T*_ and ACC_*o*_ at each time-point using eq. 5. The lowest prediction accuracy, specifically the minimum prediction accuracy (ACC_*min*_) throughout all time-points, was selected from ACC_*T*_ and ACC_*o*_ at each time-point. To access the specificity and the sensitivity at each time-point, the receiver operating characteristic (ROC) curves, the area under the ROC curve (AUC), and the 95% confidence intervals were calculated.

Second, bootstrap sampling was performed to evaluate the prediction accuracies of selected genes at time-points which had not been selected for model construction^36^. Bootstrap sampling selected individual samples from all samples by random sampling with replacement. A prediction model was constructed using selected samples and selected genes by either the proposed or conventional method. Prediction accuracies were calculated by each data set at different time-points; and this was then repeated. Finally, the mean and standard deviation were calculated using the prediction accuracies for each subset at each different time-point. The difference in the prediction accuracies between the conventional method and the proposed method was tested using the Student’s *t*-test.

Last, the differences between good and poor responders in the expression levels of the genes obtained by the proposed method were investigated. The expression levels of these genes at each time-point were classified into two groups according to the treatment response, and the differences between the two groups were tested using the Wilcoxon rank sum test. The *p* values in the Wilcoxon rank sum test were adjusted using the Benjamini-Hochberg method (BH method). Additionally, the median values of the expression levels of genes at each time-point were compared between good and poor responders to assess if the expression levels of the two groups were consistently different throughout all time-points. The names of the selected genes were obtained from Gene Cards (http://www.genecards.org/).

#### Parameter and implementation

The number of SS iterations was 500. Random sampling in SS included 12 good and 8 poor responder samples, and these were common to dataset A and B. The λ values, a hyper-parameter of SS, were changed from 0.01 to 1.00 using 0.01 increments. The threshold *θ*_*ss*_ was 0.5. The hyper-parameter of Elastic net in eq. 2 was α = 0.5, and the weight parameter in eq. 3 was *γ* = 2. The parameters of Elastic net with SS used in the conventional method were also the same as the parameters in the proposed method. The responses at 24 months after INF-β treatment in dataset A and B (Table 1) were predicted. Bootstrap sampling in the evaluation was repeated 50 times per prediction model at a different time-point, and the prediction accuracies were calculated.

For the implementation of numerical experiments, R language (ver. 3.2.5; https://cran.r-project.org/bin/windows/base/old/3.2.5/) was used, and the *limma* and *glmnet* (ver. 2.0–5) packages were used for quantile normalization and Elastic net, respectively. The source codes are available upon request.

## Results

### Comparing the proposed method to the conventional method

The proposed and conventional methods were evaluated by the analyses of datasets A and B. The genes showing the most ideal ACC_*mean*_ were identified for each dataset using both the proposed and the conventional method. The prediction accuracies at each time-point and their mean from the first evaluation are listed at Tables 2 and 3.

**Table 2:** Accuracy of prediction models by the proposed method and conventional methods with dataset.

Method	Accuracy [%]			
	t1	t2	t3	Mean (ACC_mean_)
Proposed method	(88)	***92***	79	***86***
Conventional method	(100)	71	79	83
	67	(100)	***92***	***86***
	54	83	(100)	79

**A**. Values in () were calculated by leave-one-out at the time-point of data used by the prediction model. “***Bold accuracy***” indicates the top accuracy at each time-point, but top accuracy of t1 was not presented as gene expression data at t1 was used data by the proposed method. “Accuracy” was the minimum accuracy (ACC_min_) of each method.

**Table 3:** Accuracy of prediction models by the proposed method and conventional methods with dataset B.

Method	Accuracy [%]					
	t1	t2	t3	t4	t5	Mean (ACC_mean_)
Proposed method	(96)	72	**92**	**84**	**76**	**84**
Conventional method	(92)	68	84	76	64	77
	72	(84)	76	60	64	71
	72	**92**	(96)	80	64	81
	72	64	68	(100)	40	69
	68	60	76	72	(96)	74

Values in () were calculated by leave-one-out at the time-point of data used by the prediction model. “***Bold accuracy***” indicates the top accuracy of each time-point, but top accuracy of t1 was not presented as gene expression data at t1 were used by the proposed method. “Accuracy” indicates the minimum accuracy (ACC_min_) of each method.

As an analytical result of dataset A, the proposed method identified 11 genes and constructed the prediction model using the *t1* data. With the conventional method, prediction models were constructed using the *t1*, *t2*, and *t3* data, from which 9, 8, and 21 genes were identified, respectively. Table 2 showed the prediction accuracies at each time-point and their mean. The ACC_*mean*_ and ACC_*min*_ values using the proposed method were 86% and 79%, respectively. From the conventional method using *t2* data, the ACC_*mean*_ was 86% and was comparable to that from the proposed method. The ACC_*min*_ obtained by the conventional method using the *t2* data was, however, only 67%, which was lower than that obtained by the proposed method. The ACC_*mean*_ obtained by the conventional method using the *t1* and *t3* data was 83% and 79%, respectively. The ACC_*mean*_ from the proposed method was higher than that of the conventional method. Here, we focus on the results at different time-points in the first evaluation. The prediction accuracies generated by the proposed method were 92% at *t2* and 79% at *t3*. The conventional method using *t2* data could predict treatment responses at *t3* with 92% accuracy; however, all other results were lower than those from the proposed method.

As a result of the use of dataset B, the proposed method identified 8 genes and constructed the prediction model using the *t1* data. The conventional method identified 5, 19, 7, 6, and 19 genes using *t1*, *t2*, *t3*, *t4*, and *t5* data, respectively. Table 3 lists the prediction accuracies at each time-point and the ACC_*mean*_. The ACC_*mean*_ and ACC_*min*_ of the proposed method were 84% and 72%, respectively. The ACC_*mean*_ values were 77%, 71%, 81%, 69%, and 74% using *t1*, *t2*, *t3*, *t4*, and *t5* data for model construction by the conventional method, respectively. The ACC_*min*_ values were 64%, 60%, 64%, 40%, and 60% using *t1*, *t2*, *t3*, *t4*, and *t5* data for model construction by the conventional method, respectively. The ACC_*mean*_ and ACC_*min*_ values of the proposed method were higher than those of the conventional method. We focus on the results from different time-points in the first evaluation. The prediction accuracies of the proposed method using *t1* data were 92%, 84%, and 76% at time-points *t3*, *t4*, and *t5*, respectively. The prediction accuracy at *t2* by the conventional method using *t3* data was 92%, which was higher than that obtained from the proposed method. The other accuracies generated by the proposed method were higher than those of the conventional method, with the exception of one case.

**Figure 3:**
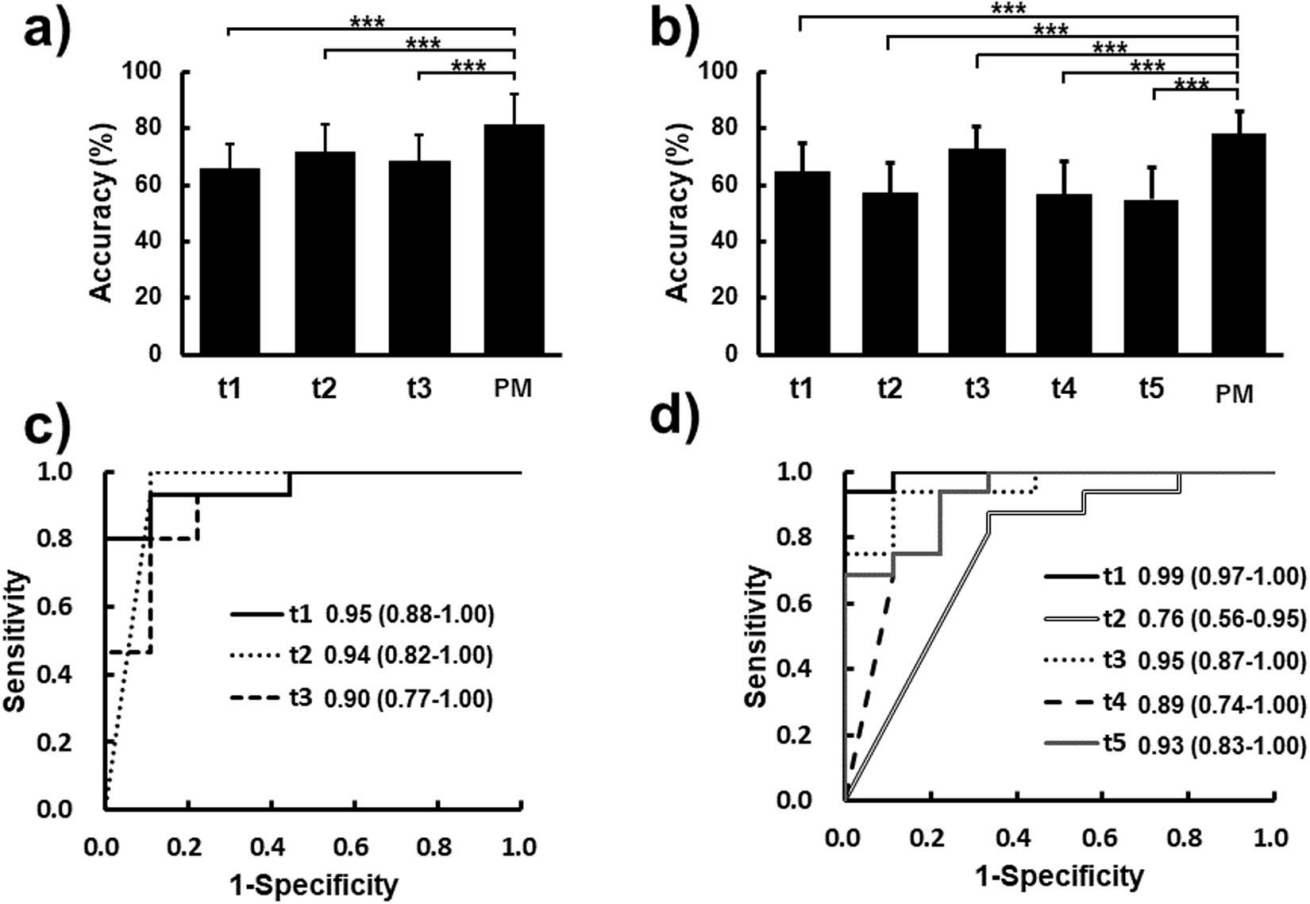
Prediction accuracies and ROC curves obtained by bootstrap sampling. (**a**) Prediction accuracies for dataset A. The accuracies are the mean accuracies of different time-points (TPs) obtained without using the prediction model. (**b**) Prediction accuracies for dataset B. As with (**a**), the accuracies are the mean accuracies. (**c**) ROC curve generated by the proposed method (PM) at each time-point of dataset A. The AUC and 95% confidence interval (CI) were calculated by ROC curves at each time-point. (**d**) ROC curve generated by PM at each time-point of dataset B. As with (**b**), the AUC and 95% CI were calculated.

Bootstrap sampling was performed to evaluate the prediction accuracies at different time-points in the second evaluation. Figure 3 shows the mean and standard deviation of prediction accuracies given by the proposed and conventional methods at different time-points. As shown in Fig. 3a, in dataset A the mean accuracy of the different time-points (*t2* and *t3*) was 81%. This prediction accuracy was significantly higher than 65% (p = 2.06 × 10^−23^), 71% (p = 1.48 × 10^−10^), and 68% (p = 1.16 × 10^−16^) at *t1*, *t2*, and *t3* in the conventional method (*p* < 0.001), respectively. As shown in Fig. 3b, in dataset B the mean accuracy of the different time-points (*t2*, *t3*, *t4*, and *t5*) provided by the proposed method was 78%. The prediction accuracies given by the conventional method at *t1*, *t2*, *t3*, *t4*, and *t5* were 64% (p = 2.41 × 10^−40^), 57% (p = 1.73 × 10^−94^), 73% (p = 8.70 × 10^−11^), 56% (p = 1.30 × 10^−103^), and 55% (p = 1.46 × 10^−78^), respectively. In dataset B, the mean accuracy of the different time-points given by the proposed method was significantly higher than those by the conventional method (*p* < 0.001). Therefore, the prediction accuracies at different time-points obtained using the proposed method were significantly higher than those given by the conventional method.

To assess the sensitivity and specificity of the prediction model of the proposed method, ROC curves and AUC in datasets A and B were measured (Fig. 3c,d). As shown in Fig. 3c, in dataset A the AUCs at *t1*, *t2*, and *t3* were 0.95, 0.94, and 0.90 given by the proposed method, respectively, and all of these were higher than or equal to 0.9. The lower limits of the 95% confidence interval were 0.88, 0.82, and 0.77 at *t1*, *t2*, and *t3*, respectively. As shown in Fig. 3d, in dataset B the AUCs at *t1*, *t2*, *t3*, *t4*, and *t5* were 0.99, 0.76, 0.95, 0.89, and 0.93, respectively. The lower limits of the 95% confidence interval were 0.97, 0.56, 0.87, 0.74, and 0.83 at *t1*, *t2*, *t3*, *t4*, and *t5*, respectively. In dataset B, the AUC and the lower limits of the 95% confidence interval of the proposed method at *t2* were 0.76 and 0.56, which were lower than or equal to the other time-points as obtained by the conventional method (Figs S1 and S2). The AUC and lower limits of the 95% confidence interval of the proposed method were the highest in almost every case.

### Selected genes by the proposed method

**Figure 4:**
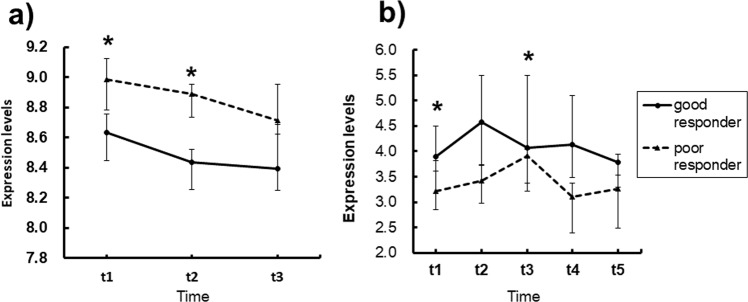
Gene expression levels of good and poor responders at each time-point. Expression levels of *HPS5* in dataset A (**a**) and *CDH2* in dataset B (**b**). Wilcoxon rank sum test. *FDR-corrected *p* < 0.05.

Eleven genes were identified in dataset A using the proposed method (Table 4) and eight genes were identified in dataset B using the proposed method (Table 5). These genes were expected to exhibit consistently higher expression levels of either good or poor responders at each time-point. The median levels of 9 genes in dataset A were consistently differentiated throughout all time-points (Table 4). In particular, the expression levels of the *HPS5* gene in poor responders at *t1* and *t2* were significantly higher than those in good responders (*p* < 0.05) (Fig. 4a). The median levels of 6 genes at each time-point were consistently higher in either group in dataset B (Table 5). In particular, the expression levels of the *CDH2* gene in good responders at *t1* and *t3* were significantly higher than those in poor responders (*p* < 0.05) (Fig. 4b). Given this, the proposed method identified a number of genes where the expression levels were consistently different throughout all time-points.

**Table 4:** Identified genes of dataset A by the proposed method.

Gene symbol	Gene name	P value			Higher GE levels at all time-points
		t1	t2	t3	
*ZBTB16*	Zinc Finger and BTB Domain Containing 16	0.064	**0**.**013**	0.137	good
*ZFP37*	ZFP37 Zinc Finger Protein	0.070	0.220	**0**.**013**	—
*HPS5*	HPS5, Biogenesis of Lysosomal Organelles Complex 2 Subunit 2	**0**.**013**	**0**.**013**	0.084	poor
*HOPX*	HOP Homeobox	0.105	**0**.**005**	0.090	good
*ARFGAP3*	ADP Ribosylation Factor GTPase Activating Protein 3	**0**.**013**	0.162	0.105	good
*CALML5*	Calmodulin Like 5	0.077	**0**.**013**	0.126	good
*VPS26A*	VPS26, Retromer Complex Component A	**0**.**026**	0.090	0.205	good
*SLC5A4*	Solute Carrier Family 5 Member 4	0.190	**0**.**022**	0.190	good
*MBL2*	Mannose Binding Lectin 2	0.149	**0**.**013**	0.640	—
*DLGAP4*	DLG Associated Protein 4	**0**.**007**	0.115	0.390	good
*CACNA1C*	Calcium Voltage-Gated Channel Subunit Alpha1 C	0.064	0.382	0.390	poor

P values were adjusted using the BH method, and “**Bold accuracy**” exhibited significantly different gene expression (GE) levels between good and poor responders (p < 0.05). If GE levels of good responders at each gene were higher than those of poor responders at all time-points (TPs), “good” was represented in the final column.

**Table 5:** Identified gene list of dataset B by the proposed method.

Gene symbol	Gene name	P value					Higher GE levels of all TPs
		t1	t2	t3	t4	t5	
*SMA4*	Survival of Motor Neuron 1, Telomeric	0.072	0.250	**0**.**009**	0.082	0.082	good
*MIR7114_NSMF*	MicroRNA 7114/NMDA Receptor Synaptonuclear Signaling and Neuronal Migration Factor	0.072	0.082	**0**.**005**	0.130	0.314	good
*LSM8*	LSM8 Homolog, U6 Small Nuclear RNA Associated	0.452	**0**.**009**	0.082	0.082	0.441	—
*FLAD1*	Flavin Adenine Dinucleotide Synthetase 1	0.071	**0**.**009**	0.344	0.056	0.072	poor
*RRN3P1*	RRN3 Homolog, RNA Polymerase I Transcription Factor Pseudogene 1	0.419	0.179	0.082	0.082	**0**.**023**	poor
*RASL10A*	RAS Like Family 10 Member A	**0**.**033**	0.334	0.344	0.452	0.314	—
*IER3IP1*	Immediate Early Response 3 Interacting Protein 1	0.115	0.072	**0**.**005**	0.216	0.082	poor
*CDH2*	Cadherin 2	0.250	**0**.**033**	0.397	**0**.**043**	0.082	good

P values were adjusted using BH method, and “**Bold accuracy**” represents significantly different gene expression (GE) levels between good and poor responders (p < 0.05). If GE levels of good responders at each gene were higher than those of poor at all time-points (TPs), “good” was represented in the final column.

## Discussion

The genes identified by the proposed method showed consistent differentiation throughout all time-points and accurately predicted the responses of MS patients to INF-β treatment.

The ACC_*mean*_ and ACC_*min*_ values given by the proposed method in dataset A were 86% and 79%, respectively. The ACC_*mean*_ value was equal to or higher than that given by the conventional method (Table 2). The prediction model obtained from the conventional method using *t2* data had a nearly identical ACC_*mean*_ value to that given by the proposed method; however, the ACC_*min*_ value of the proposed method was higher than that of the conventional method. The ACC_*mean*_ and ACC_*min*_ values of the proposed method in dataset B were 84% and 72%, respectively (Table 3). These values were higher than those of the conventional method. Thus, the proposed method yielded higher and more accurate predictions throughout most time-points in comparison to those given by the conventional method (Tables 2 and 3). Additionally, the prediction accuracies at different time-points were evaluated by bootstrap sampling. Figure 3a,b provide the means and standard deviations of the prediction accuracies of different time-points calculated by bootstrap sampling. The mean accuracy of different time-points by the proposed method was 81%, which is higher than those obtained by the conventional method (Fig. 3a). This result indicates that the proposed method could achieve significantly higher prediction accuracies than the conventional method at different time-points. In dataset B, the mean accuracy of different time-points was 78%, and this was significantly higher than those given by the conventional method (Fig. 3b). Additionally, SES algorithm analysis of the static-longitudinal scenario is used as a conventional method^22^, and this is compared with our proposed method. The static-longitudinal scenario in this method is expected to identify genes showing consistent differentiation throughout all time-points^22^. Using the procedures of the proposed method (Fig. 2d), the ACC_*mean*_ of the genes identified by SES algorithm was calculated, and a prediction model was created. The prediction accuracies at different time-points using SES algorithm were calculated by bootstrap sampling (Table S1). These mean accuracies obtained from our proposed method were higher than those given by this conventional method. Therefore, the proposed method using time-course data could achieve a high prediction accuracy compared with those provided by the conventional methods. Given this, the proposed method provided higher accuracy throughout all time-points.

Figure 3c,d show the sensitivity and specificity of the proposed method; and AUC was approximately 0.90 at most time-points in both datasets A and B. The AUC at *t2* in dataset B given by the proposed method, however, was 0.76, which was lower than the AUC at other time-points and equivalent to the conventional method, as shown in Figs S1 and S2. The results at each time-point (Tables 2 and 3 and Fig. S3) revealed that the prediction accuracies did not depend upon the order of the time-course sampling, and the prediction accuracies by the proposed method were high at most time-points. There was, however, a case where the prediction accuracy was lower than that of the conventional method.

As shown in Tables 4 and 5, most genes from the proposed method showed different expression levels consistently throughout all time-points. Changes in those levels differentiated between good and poor responders consistently throughout the time-courses significantly (Fig. 4). Given this, the proposed method identified genes showing consistent differentiation throughout multiple time-points and could differentiate between good and poor responders.

The proposed method did not identify identical genes between datasets A and B. For dataset A, associations between MS and *ZBTB16* and *HOPX* were reported^36^. *Th17* cells are a subset of T helper cells involved in several immune diseases, including MS. *ZBTB16* was reported to activate differentiation of *Th17* cells, and this contributed to the maintenance of the phenotype of *Th17* cells in the human body^37^. In regard to the relationship between the functional defects of *T* cells and autoimmune encephalomyelitis, many experiments and reviews reported the deletion of the *HOPX* gene as responsible for decreasing suppressor ability of pTreg cells^38,39^. For dataset B, there were reports detailing *CDH2*^39^. Microglia, a type of glial cell of the central nervous system, are known as central immunocompetent cells, and *CDH2* is involved in the context of these cells^40^. Many genes identified in dataset A were related to cancer, but their association with MS remains unclear.

The proposed method possessed several limitations. First, there were time-points where the prediction accuracy given by the proposed method was lower. Second, the γ value as the weights of Elastic nets must be set adequately to ensure accurate prediction. Finally, we used genes as independent variables; however, the interactions of genes could also be considered as explanatory variables to obtain higher accuracy predictions.

INF-β treatment is known to be effective in the prevention of relapses of MS; however, the accurate prediction of INF-β treatment responses is still necessary to solve the problem of individual variations and the side effects associated with treatment. Microarray has been used to identify genes for predicting treatment responses. There are several difficulties, however, associated with microarray analysis, including a high dimensionality and multicollinearity. Additionally, the conventional method only allows data analysis at a single time-point. Therefore, a new method suitable for time-course data analysis should be developed to identify genes showing highly accurate prediction abilities throughout all time-courses. Here, we proposed a method to identify genes using Elastic net accommodating time-course data. The three major features include sparse modelling to allow for the efficient identification of genes (gene numbers ≫ sample size); Elastic net utilization to prevent the multicollinearity of expression levels among genes, and Elastic net modification to identify genes showing consistent differentiation throughout the time-course.

Two publicly available datasets were used to identify genes showing highly accurate prediction abilities throughout all time-courses. The mean prediction accuracies of different time-points given by the proposed and conventional method were compared. The accuracies obtained using two datasets were 71% and 73% for the conventional method, and 81% and 78% (significantly higher) for the proposed method. The proposed method identified 11 and 8 genes in the two datasets. Differences in the expression levels of 9 and 6 genes between good and poor responders were consistent throughout the data at all time-points. Therefore, the genes identified by the proposed method were identified as capable of high-accuracy prediction throughout multiple time-points. Additionally, these genes included genes previously reported to be related to MS. The proposed modified Elastic net method for the time-course data analyses was used to identify genes showing consistent differentiation between two outcome groups throughout time-courses. Here, we demonstrated the use of this modified Elastic net for the prediction of INF-β treatment responses in patients with MS. Additionally, this method could also be used for microarray time-course data analyses.

## Supplementary information

Supplyment information
