# Supplementary material for: Elastic net-based prediction of IFN-β treatment response of patients with multiple sclerosis using time series microarray gene expression profiles
Source: Sci Rep. 2019 Feb 12;9:1822. doi: 10.1038/s41598-018-38441-2 (PMC6372673; doi:10.1038/s41598-018-38441-2)
Supplement: Supplementary file 1 — Supplyment information [file 41598_2018_38441_MOESM1_ESM.docx]

**Supplementary Information for**

**Elastic net-based prediction of IFN-β treatment response of patients with multiple sclerosis using time series microarray gene expression profiles**

Arika Fukushima^1^, Masahiro Sugimoto^234^, Satoru Hiwa^1^, Tomoyuki Hiroyasu^1*^

^1^Doshisha University, Graduate School of Life and Medical Sciences, Kyoto, Japan

^2^Research and Development Center for Minimally Invasive Therapies Health Promotion and Preemptive Medicine, Tokyo Medical University, Shinjuku, Tokyo 160-8402, Japan

^3^Institute for Advanced Biosciences, Keio University, Tsuruoka, Yamagata 997-0052, Japan

^4^University of Tsukuba, Research and Development Center for Precision Medicine, Tukuba, Ibaraki, 305-8550, Japan

**Corresponding to:** Tomoyuki Hiroyasu, Ph.D., Professor

Faculty of Life and Medical Sciences, Doshisha University, Kyotanabe-shi, Kyoto, 610-0321, Japan

TEL 0774-65-6932, FAX 0774-65-6019

E-mail: tomo@mis.doshisha.ac.jp

**Running title:** Time course-based Elastic net

**Keywords**: Time-course data, Elastic net, Multiple sclerosis, prediction

**Table S1 Selected genes and accuracy by bootstrap sampling using SES algorithm.**

| Dataset name | Dataset A | Dataset B |
| --- | --- | --- |
| Identify gene symbol | BID | CTDSPL |
| time point of data for prediction model | t1 | t5 |
| Mean accuracy by bootstrap sampling [%] | 57 (p=2.32×10^64^) | 61 (p=2.92×10^32^) |

Values in () are p-values of comparison between the SES algorithm and our proposed method. For the implementation of the SES algorithm, the MXM package was used in R.

**Figure S1 ROC of prediction model given by the conventional method in dataset A.** Prediction model using *t1* **(a),** *t1* **(b)**, and *t1* **(c)** data.

**Figure S2 ROC of prediction model given by the conventional method in dataset B.** Prediction model using at *t1* **(a)**, and *t2* **(b)**, *t3* **(c)**, *t4* **(d)**, and *t5* **(e)** data.

**Figure S3 Prediction accuracies obtained using the proposed method (PM) and conventional method at each time point using bootstrap sampling.** Prediction accuracies of each model at *t2* **(a)** and *t3* **(b)** data in dataset A, *t2* **(c)**, *t3* **(d)**, *t4* **(e)**, and *t5* **(f)** data in dataset B.
